# Kids SIP*smart*ER reduces sugar-sweetened beverages among Appalachian middle-school students and their caregivers: a cluster randomized controlled trial

**DOI:** 10.1186/s12966-024-01594-7

**Published:** 2024-04-25

**Authors:** Jamie M. Zoellner, Wen You, Kathleen Porter, Brittany Kirkpatrick, Annie Reid, Donna Brock, Phillip Chow, Lee Ritterband

**Affiliations:** 1 Department of Public Health Sciences, University of Virginia, UVA Cancer Center Research and Outreach Office, 16 East Main Street, Christiansburg, VA 24073 USA; 2 Department of Public Health Sciences, University of Virginia, 560 Ray C Hunt Drive, Charlottesville, VA 22908 USA; 3 Department of Psychiatry and Neurobehavioral Sciences, University of Virginia, 560 Ray C Hunt Drive, Charlottesville, VA 22908 USA

**Keywords:** Sugar sweetened beverages, Research design, Behavioral research, Randomized controlled trial, Rural population, Health literacy

## Abstract

**Background:**

High consumption of sugar-sweetened beverages (SSB) is a global health concern. Additionally, sugar-sweetened beverage (SSB) consumption is disproportionately high among adolescents and adults in rural Appalachia. The primary study objective is to determine the intervention effects of Kids SIP*smart*ER on students’ SSB consumption. Secondary objectives focus on caregivers’ SSB consumption and secondary student and caregiver outcomes [e.g, body mass index (BMI), quality of life (QOL)].

**Methods:**

This Type 1 hybrid, cluster randomized controlled trial includes 12 Appalachian middle schools (6 randomized to Kids SIP*smart*ER and 6 to control). Kids SIP*smart*ER is a 6-month, 12 lesson, multi-level, school-based, behavior and health literacy program aimed at reducing SSB among 7th grade middle school students. The program also incorporates a two-way text message strategy for caregivers. In this primary prevention intervention, all 7th grade students and their caregivers from participating schools were eligible to participate, regardless of baseline SSB consumption. Validated instruments were used to assess SSB behaviors and QOL. Height and weight were objectively measured in students and self-reported by caregivers. Analyses included modified two-part models with time fixed effects that controlled for relevant demographics and included school cluster robust standard errors.

**Results:**

Of the 526 students and 220 caregivers, mean (SD) ages were 12.7 (0.5) and 40.6 (6.7) years, respectively. Students were 55% female. Caregivers were mostly female (95%) and White (93%); 25% had a high school education or less and 33% had an annual household income less than $50,000. Regardless of SSB intake at baseline and relative to control participants, SSB significantly decreased among students [-7.2 ounces/day (95% CI = -10.7, -3.7); *p* < 0.001, effect size (ES) = 0.35] and caregivers [-6.3 ounces/day (95% CI = -11.3, -1.3); *p* = 0.014, ES = 0.33]. Among students (42%) and caregivers (28%) who consumed > 24 SSB ounces/day at baseline (i.e., high consumers), the ES increased to 0.45 and 0.95, respectively. There were no significant effects for student or caregiver QOL indicators or objectively measured student BMI; however, caregiver self-reported BMI significantly decreased in the intervention versus control schools (*p* = 0.001).

**Conclusions:**

Kids SIP*smart*ER was effective at reducing SSB consumption among students and their caregivers in the rural, medically underserved Appalachian region. Importantly, SSB effects were even stronger among students and caregivers who were high consumers at baseline.

**Trial registration:**

Clincialtrials.gov: NCT03740113. Registered 14 November 2018– Retrospectively registered, https://clinicaltrials.gov/ct2/show/NCT03740113.

## Background

Reducing sugar-sweetened beverages (SSB, e.g., soda/pop, sweet tea and coffee, sports and energy drinks, fruit drinks) is a key public health priority in the United States (U.S.) and globally [1, 2]. SSB consumption exceeds recommendations in many high-income countries, and with widespread urbanization, SSB are also increasing in many low- and middle-income countries and with indication [3–5]. In the U.S., SSB are the largest single source of added sugar, top source of energy, and contribute approximately 8% and 7% of total energy intake among youth and adults, respectively [3, 6]. Unfortunately, disparities persist in SSB consumption patterns. For example, excessive SSB consumption is well-documented among low socioeconomic, low health literate, and racial and ethnic minorities [7–16]. Differences across the life span are also apparent, with the highest SSB consumption among adolescents 12–19 years of age and adults 20–39 years of age [6, 9, 12, 17]. Finally, daily SSB intake is also significantly higher in nonmetropolitan U.S. counties, relative to metropolitan counties (adjusted prevalence ratio = 1.32) [7].

These disparities in SSB consumption patterns are concerning given the strong and consistent evidence, in both youth and adults, linking SSB to chronic health conditions like obesity, dental erosion and decay, diabetes, cardiovascular disease, and hypertension [18–28]. Given these negative health consequences, it is important to intervene on SSB behaviors from both a primary and secondary prevention perspective [29]. Specifically, for individuals meeting SSB recommendations, efforts are needed to prevent an increase in SSB consumption. Among individuals not meeting SSB recommendations, the goal should be to move them closer to recommendations. Prevention efforts targeting adolescence is particularly critical since there is increased autonomy in food and beverage choices as well as habit formation during this life-span period [30, 31]. It may be equally as important to engage caregivers, who serve as SSB role models and home environment gatekeepers [32–39].

As evidenced by several systematic reviews, intervention literature pertaining to reducing SSB consumption among youth is mixed [40–42]. One systematic review of 36 school-based intervention trials targeting adolescents found promising results for those classified as either educational/behavioral or legislative/environmental [40]. Yet, interpretations are limited due to different study designs [e.g., only 13 (36%) were randomized controlled trials (RCT)], absence of validated SSB measures [e.g., only 10 (28%) used validated SSB measures], and lack of meta-analyses [40]. Another meta-analysis of 28 RCTs targeting children and adolescents found a promising average SSB effect size (ES) of 0.48 [41]. These reductions were even greater when interventions included techniques related to role-modeling or were delivered in home settings [41]. However, only 5 of 28 studies focused on adolescents, and the average SSB ES of these interventions was much smaller (i.e., 0.05). A third meta-analysis of 19 RCT and pre-post studies targeting socioeconomically disadvantaged ethnic minority adolescents revealed no significance between group differences in reduction of SSB intake [43]. Finally, a systematic review of 55 studies in children and adolescents examined SSB reduction strategies across the socio-ecological model. It was concluded that existing literature provided insufficient evidence to inform translation into real-world settings, consequently limiting the potential public health impact of existing SSB interventions [42].

Collectively these reviews highlight the breadth of available SSB reduction interventions and programs, yet gaps and opportunities remain. First, there is a clear need for theory-driven behavioral interventions assessed using trial designs that maximize both internal and external validity and that use validated SSB measures [40, 42]. Second, while schools seem to be a promising setting for SSB reduction interventions, few studies have focused on middle school students in the U.S., and substantially fewer on middle school students in U.S. rural regions [40–42]. Third, most interventions target SSB reduction as a part of a general healthy eating objective [40]. Interventions rarely target SSB specifically [41], even though this approach is more effective [44]. Finally, SSB reduction studies for middle school students rarely include a caregiver component [40–42, 45] and few known studies have used a scalable text message strategy to engage middle school caregivers [46].

The need for targeted behavioral SSB strategies is especially pronounced in at-risk, rural, and underserved U.S. regions where SSB intake is disproportionately high, such as Appalachia [47–49]. Most Appalachian counties are federally designated as medically underserved, including healthcare provider shortage areas [50]. Additional challenges include transportation issues, geographical isolation, and widespread poverty [51, 52]. Given these challenges and lack of access to evidence-based prevention programs [53, 54], schools may provide the best opportunity to reach the largest and most representative sample of adolescents in underserved rural regions like Appalachia. The narrowing digital divide (e.g., expanding cellular network infrastructure and mobile phone ownership) [55, 56] in rural regions and growing evidence on text message interventions [57–59] may provide a unique opportunity to engage adolescent caregivers using a text message platform.

Kids SIP*smart*ER is a primary prevention intervention conceptualized to address identified literature gaps and to meet the needs of rural Appalachian school systems. Working in partnership with schools and targeting both middle school students and their caregivers, the overarching goal of Kids SIP*smart*ER was to decrease SSB consumption and to ultimately reduce SSB-related health inequities and chronic conditions in rural Appalachia. Because Kids SIP*smart*ER was designed to be delivered to groups of students at different schools, the study is a cluster randomized controlled trial (RCT) whereby the randomized occurred at the school level. The primary objective of this cluster RCT is to examine 0–7 month effectiveness on SSB among students receiving Kids SIP*smart*ER, as compared to students at control schools. Relative to control students, it was hypothesized that students receiving Kids SIP*smart*ER would demonstrate greater SSB reductions and between condition effects would be larger among students who were higher SSB consumers at baseline. Secondary objectives are to examine (1) SSB effects among caregivers, (2) other secondary student and caregiver outcomes [i.e., body mass index (BMI), quality of life (QOL), self-reported health], and (3) program implementation fidelity.

## Methods

This cluster RCT included a convenience sample of 12 Appalachian middle schools in southwest Virginia and southeastern West Virginia; complete protocol details are previously published [60]. To allow for management of research resources and retention of schools from the point of recruitment to trial initiation, 12 schools were recruited and randomized within three separate blocks (i.e., 2018–2019, 2019–2020, and 2021–2022 academic years). COVID-19 impacted the trial, including but not limited to school shutdowns beginning in spring 2020 that halted enrollment of block 3 schools during the 2020–2021 academic year. Within each block, simple randomization was used with two schools each randomized to intervention and control conditions. Students and caregivers were blinded to condition allocation status. After the first year, control schools received the Kids SIP*smart*ER intervention (i.e., transitioned to delayed intervention condition). The larger multi-level trial is a type 1 hybrid design and is guided by the RE-AIM (***r***each, ***a***doption, ***e***ffectiveness, ***i***mplementation, and ***m***aintenance) framework. This primary outcome paper focuses on student and caregiver ***e***ffectiveness data from each school’s first year of trial participation as well as ***i***mplementation fidelity.

This study was approved by the University of Virginia Institutional Review Board (protocol number 2371). Superintendents and middle school principals were informed of the study approach and agreed to randomization and data collection procedures and to support teachers’ facilitation of curriculum implementation. Intervention and control schools received $1500 and $1000, respectively, during the first year of trial participation. Caregivers provided consent and students provided assent. Students who returned signed consent forms, regardless of consent status, received a nominal prize. At the 7-month post-program assessment, students received a t-shirt. Caregivers received a $10 gift card each time they returned a survey. Because Kids SIP*smart*ER was implemented during a regular classroom period, all students participated, regardless of consent or assent status. Yet, data was analyzed only if caregiver consent and student assent were provided.

This cluster RCT was conservatively powered based on a 7-month ES of 0.3, a 0.05 type I error, and a 0.01 interclass correlation of students’ SSB intake [60]. To achieve 80% power under these assumptions, a total of 12 schools/clusters (6 schools per condition) were needed, with 54 enrolled students per school (and 49 retained after an anticipated 10% attrition rate at 7-month).

### Eligibility, recruitment, and enrollment

School eligibility criteria included: (1) location in central Appalachia, (2) approximately 80–200 students in 7th grade, and (3) an 8th grade within the same 7th grade school building to facilitate collection of maintenance data. Within participating schools, all 7th grade students were eligible to participate, regardless of SSB consumption. Similarly, one caregiver per 7th grade student was eligible to participate, regardless of SSB consumption. Caregivers could choose to consent their child only or consent both their child and their selves. Enrollment in the study required consent and assent, along with baseline assessment completion.

At each school, recruitment efforts included strategies based on previously successful recruitment efforts [61, 62], including: (1) an initial recruitment packet distributed to caregivers (i.e., informational letter signed by the school’s principal, a study flyer, and a consent form), (2) redistribution of additional consent forms, and (3) a personalized phone call to remind caregivers to return consent forms and to answer study-related questions, if needed. Additional recruitment strategies were customized to the needs of each school (e.g., robo-calls or email/app blasts to inform families about Kids SIP*smart*ER; information provided by research team members attending “Back-to-School Nights”).

### Intervention description and implementation

Kids SIP*smart*ER was adapted from the evidence-based SIP*smart*ER intervention targeting Appalachian adults [49, 63–74]. The intervention was also informed by formative and pilot testing phases among Appalachian middle school students and caregivers [47, 75, 76]. Specifically, Kids SIP*smart*ER is guided by the Theory of Planned Behavior (TPB) and also integrates skill-based health literacy concepts (e.g., numeracy, media literacy, and public health literacy). It is a multi-level, 6-month, school-based, behavioral intervention aimed at reducing SSB among 7th grade middle school students. The intervention also incorporates a text messaging strategy to engage caregivers in SSB role modeling and to support improvements in SSB practices, rules, and home environment. Complete intervention content, theoretical foundations, behavioral change techniques, and implementation details are published elsewhere [60, 77].

#### Student component

 In brief, 12 classroom-based, face-to-face lessons were designed to fit within a 40–50 min class period and intended for nine and three lessons delivered in fall and spring, respectively. Using a drink traffic light system, students were educated on sugary and non-sugary drinks. Lessons 1–6 focus on making personal changes, lessons 7–9 emphasize encouraging change in the community, and lessons 10–12 focus on motivating and maintaining changes. Students received a workbook that included worksheets related to core educational and behavioral content application, action plan development, goal setting, and SSB self-monitoring.

#### Caregiver component

 The Qualtrics Research Suite, hosted by the University of Virginia, was used to program personalized text message logic, deliver messages, and temporarily store secure data. Researchers managed all aspects of the text message intervention. Caregivers received an initial SSB-related newsletter followed by approximately two text messages per week in the fall and two text messages per month in the spring. Combinations of text messages included: (1) *assessment messages* (two-way): every 4–5 weeks in which caregivers reported daily SSB frequency over the past week for themselves and their child and received personalized feedback on progress, (2) *personalized strategy messages* (two-way and one-way): relevant to current barriers, during assessments caregivers chose the type of tailored one-way strategy messages they wanted to receive over the subsequent 4–5 weeks (e.g., parenting tips, tasty alternatives, breaking habits, home and shopping tips, and dealing with friends and family), and (3) *educational messages* (one-way): non-tailored messages that paralleled their child’s classroom lessons, with about half in an infographic form.

#### Teacher component

 Kids SIP*smart*ER also included a teacher implementation strategy that consists of professional development, technical assistance, and a secure website that contains all student curriculum and teacher training resources. In each school’s first year of implementation, Kids SIP*smart*ER was co-delivered by master’s degree level researchers and by teachers. Though beyond the scope of this manuscript, after the first year of implementation, teachers were trained and received technical assistance to deliver Kids SIP*smart*ER with reduced in-class support from researchers.

### Data Collection methods

Data was collected at baseline and 7-months (immediately post-program). Student data was collected at school during the school day, via paper and pencil methods. Researchers read survey items aloud while students followed along and filled in their responses. Disruptions caused by COVID-19 in spring 2020 triggered transition to a secure on-line survey format for students at one school. For caregivers, paper and pencil survey packets were sent home from schools prior to COVID-19. However, COVID-19 disruptions triggered transition to a secure on-line survey format, sent via text message. As further detailed below, questionnaires were similar for students and caregivers (i.e., beverages, quality of life, self-rated health).

### Student measures

#### Beverage behaviors

 Primary beverage behavior outcomes were assessed using an adapted version of the validated Beverage Intake Questionnaire (BEVQ-15) [78–80]. The five questions computing amounts of SSB (i.e., regular soft drinks, sweetened juice beverage/drink, sweetened tea, coffee with sugar, energy/sports drinks) were not altered. For each beverage question, consumption frequency ranged across seven response categories from never or less than 1 time per week to 3 or more times per day. Portion sizes ranged across six response categories from 6 ounces or less to greater than 20 ounces. When greater than 20 ounces was selected, an open text field queried respondents to write in exact ounces. Using standardized and validated scoring procedures, daily totals for each beverage were determined by multiplying intake frequency by portion size [78–80]. Likewise, the five categories of SSB were summed to obtain total daily intake of all SSB.

#### Quality of life (QOL) and self-rated health

 Previously validated instruments and scoring procedures were used to evaluate QOL and self-rated health [81, 82]. Among students, school-related QOL was assessed with the 5-item school functioning subscale of the Pediatric QOL Inventory which used a 5-point Likert scale (i.e., 1 = never a problem, 5 = almost always a problem) [81]. Applying validated scoring procedures, items were reverse-scored and linearly transformed to a 0 to 100 scale with higher scores indicating higher school-related QOL [81, 83]. Self-rated health was assessed using a single-item question from the Youth Risk Factor Behavior Surveillance System (i.e., In general, how would you rate your overall health) reported on a 5-point Likert scale (i.e., 1 = poor to 5 = excellent) [82].

#### Body Mass Index (BMI)

For students, height and weight were measured by trained research staff using a research-grade calibrated digital DC-430U Tanita® scale and research-grade portable Seca 213 I stadiometer. Age and sex-specific CDC growth charts were used to calculate BMI percentiles, including student’s sex, weight, height, birthday, and data collection date [84]. These data were also used to calculate BMI z-scores for students.

#### Demographics

 Self-reported student demographic questions included gender, age, and race/ethnicity [82].

### Caregiver measures

#### Beverage behaviors

 Identical to the student measures, the primary beverage behavior outcomes were assessed and scored using an adapted version of the BEVQ-15, including the five SSB related questions [78–80].

#### Quality of life (QOL) and self-rated health

 The Centers for Disease Control (CDC) Healthy Days Core Module was used to assess QOL [85]. This module has two items that independently query how many days physical and mental health were not good. Using validated scoring procedures, an unhealthy days score was computed by adding the number of physically and mentally unhealthy days, with a maximum score of 30 days. This module also has an identical self-rated health item, as described above for students [85].

#### Body Mass Index (BMI)

 Caregivers self-reported their height and weight. These data were converted to BMI units and categories in accordance with CDC protocol [86].

#### Demographics

 Self-reported caregiver demographics included gender, age, race/ethnicity, education status and income [87].

### Intervention Fidelity measures

Guided by prior SSB implementation research [88], lesson specific fidelity checklists were developed and completed by research team members and teachers following each lesson delivery. Checklists assessed the degree to which specific lesson activities were completed (i.e., 1 = completed, 0.5 = partially completed, 0 = not completed), modifications (i.e., 0 = no, 1 = yes), and perceptions of student engagement (i.e., 1 = strongly disagree to 7 = strongly agree). Qualtrics reports were monitored to assess the researcher’s fidelity to sending the caregiver text messages per protocol, as well as to track non-functioning phone error codes.

### Data analysis

**Fig. 1:**
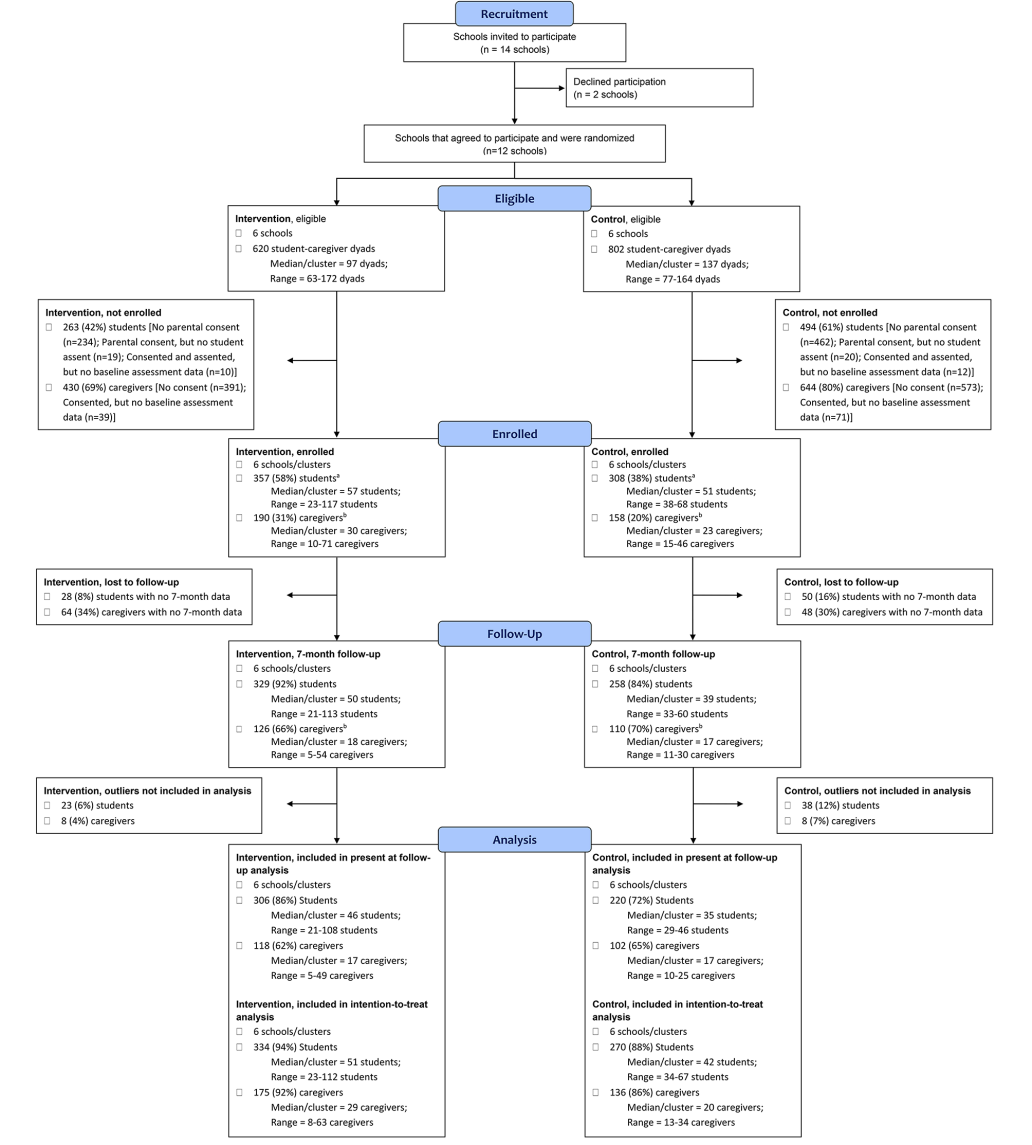
Consort flow diagram ^a^consented, assented, and baseline assessment completed ^b^consented and baseline assessment completed

Data were examined for presence of outliers, violations of normality (for continuous variables), and patterns of missing data. Outliers were identified based on interquartile range (IQR) of the 0–7-month SSB change scores. The IQR is good for identifying outliers, especially in skewed and asymmetric distributions [89]. Participants with SSB change scores ≥ 2 × IQR were marked as outliers and excluded from analysis, which is a more conversative approach than the commonly accepted ≥ 1.5 × IQR cutoff [90]. This conversative cut-point was chosen to achieve balance between conservation of the analytic sample and minimization of data noises prevalent in students’ classroom-collected survey and caregivers’ remote collected survey. Of 587 students and 236 caregivers with completed baseline and 7-month surveys, 61 (10%) and 16 (7%), respectively, were removed based on this criterion (Fig. 1). These identified SSB outliers were excluded from all secondary variable analyses, and the ≥ 2 × IQR cut-point was further applied to identify and remove outliers based on BMI, QOL, and self-reported health change scores (see sample sizes in Tables 2 and 3).

**Table 1:** Baseline demographic characteristic of enrolled students, overall and by randomized condition

	Baseline and 7-Month Completers		
	Overall *n* = 526	Kids SIPsmartER *n* = 306	Control *n* = 220
STUDENTS			
**Age** (years), M (SD)	12.7 (0.5)	12.6 (0.5)	12.7 (0.4)
**Gender**			
Female, n (%)	291 (55%)	182 (59%)	107 (48%)
Male, n (%)	229 (44%)	122 (40%)	109 (50%)
Other or unknown, n (%)	6 (1%)	2 (1%)	4 (2%)
**Race**			
Black, n (%)	20 (4%)	9 (3%)	11 (5%)
White, n (%)	456 (87%)	268 (88%)	188 (85%)
Other or unknown, n (%)	50 (9%)	29 (9%)	21 (10%)
**Ethnicity**			
Hispanic, n (%)	24 (5%)	15 (5%)	9 (4%)
**BMI [** *n* ** = 415]**			
BMI z-score, M (SD)	0.9 (1.1)	0.9 (1.1)	1.0 (1.0)
BMI percentile, M (SD)	74.1 (26.2)	73.2 (27.1)	75.8 (24.6)
Underweight (BMI < 5th percentile), n (%)	9 (2%)	5 (2%)	4 (2%)
Healthy Weight (BMI 5th - <85th percentile), n (%)	245 (47%)	142 (46%)	103 (47%)
Overweight (BMI 85th - <95th percentile), n (%)	96 (18%)	57 (19%)	39 (18%)
Obese (BMI 95th - <99th percentile), n (%)	98 (19%)	58 (19%)	40 (18%)
Severe Obesity (BMI ≥ 99th percentile), n (%)	48 (9%)	33 (11%)	15 (7%)
Other or unknown, n (%)	30 (6%)	11 (4%)	19 (9%)
**Caregiver participation in the study**			
Partial or Complete parent participation, n (%)	279 (53%)	159 (52%)	120 (55%)

Notes: M = Mean; SD = Standard Deviation

**Table 2:** Baseline demographic characteristic of enrolled caregivers, overall and by randomized condition

	Baseline and 7-Month Completers		
	Overall *n* = 220	Kids SIPsmartER *n* = 118	Control *n* = 102
CAREGIVERS			
**Age**			
(years), M (SD)	40.6 (6.7)	40.8 (7.0)	40.4 (6.2)
**Gender**			
Female, n (%)	208 (95%)	112 (95%)	96 (94%)
Male, n (%)	12 (5%)	6 (5%)	6 (6%)
**Race**			
Black, n (%)	7 (3%)	3 (3%)	4 (4%)
White, n (%)	205 (93%)	112 (95%)	93 (91%)
Other or unknown, n (%)	8 (4%)	3 (3%)	5 (5%)
**Ethnicity**			
Hispanic, % (n)	1% (2)	1% (1)	0% (0)
**Education**			
High School, GED, or less, n (%)	56 (25%)	28 (24%)	28 (27%)
Some college, Associates degree, n (%)	85 (39%)	49 (42%)	36 (35%)
4-year college degree or higher, n (%)	71 (32%)	36 (31%)	35 (34%)
Other or unknown, n (%)	8 (4%)	5 (4%)	3 (3%)
**Household Income**			
< $25,000, n (%)	38 (17%)	20 (17%)	18 (18%)
$25,000-$49,999, n (%)	36 (16%)	16 (14%)	20 (20%)
$50,000-$74,999, n (%)	43 (20%)	25 (21%)	18 (18%)
≥ $75,000, n (%)	68 (31%)	37 (31%)	31 (30%)
Other or unknown, n (%)	35 (16%)	20 (17%)	15 (15%)
**BMI**			
BMI unit, kg/m^2^, M (SD)	30.8 (7.9)	32.1 (7.7)	29.5 (8.0)
Underweight (BMI < 18.5 kg/m^2^), n (%)	4 (2%)	1 (1%)	3 (3%)
Healthy Weight (BMI 18.5–24.9 kg/m^2^), n (%)	44 (20%)	16 (14%)	28 (27%)
Overweight (BMI 25–29.9 kg/m^2^), n (%)	58 (26%)	31 (26%)	27 (26%)
Obese (BMI 30–34.9 kg/m^2^), n (%)	42 (19%)	26 (22%)	16 (16%)
Severe Obesity (BMI ≥ 35 kg/m^2^), n (%)	55 (25%)	34 (29%)	21 (21%)
Other or unknown, n (%)	17 (8%)	10 (8%)	7 (7%)
**Weight** (kg), M (SD)	85.6 (24.1)	89.2 (23.1)	81.4 (24.8)

Notes: M = Mean; SD = Standard Deviation

**Table 3:** Student outcomes: 0–7 month changes in sugar-sweetened beverages (SSB), Body Mass Index (BMI) and quality of life (QOL), by randomized treatment condition

Variable	Sample size	Kids SIPsmartER				Control				Relative effects between conditions^b^		
		Baseline^a^	7-month^a^	Adjusted change baseline to 7-month^b^		Baseline^a^	7- month^a^	Adjusted change baseline to 7-month^b^				
		Mean (SD)	Mean (SD)	Coeff (95% CI)	p- value	Mean (SD)	Mean (SD)	Coeff (95% CI)	p- value	Coeff (95% CI)	p- value	Effect size
**Sugar-sweetened beverages (SSB)**												
SSB, ounces	*n* = 526	29.6 (26.0)	19.8 (21.4)	-9.9 (-12.2, -7.6)	< 0.001	22.3 (19.5)	19.7 (19.3)	-2.7 (-5.4, -0.1)	0.045	-7.2 (-10.7, -3.7)	< 0.001	0.35
SSB, ounces; baseline consumption > 8oz	*n* = 407	35.6 (25.5)	22.3 (22.3)	-13.5 (-16.2, -10.8)	< 0.001	29.4 (18.4)	23.8 (20.2)	-5.7 (-8.5, -2.9)	< 0.001	-7.8 (-11.8 -3.9)	< 0.001	0.38
SSB, ounces; baseline consumption > 12oz	*n* = 349	39.6 (25.1)	24.2 (23.0)	-15.5 (-19.0, -12.0)	< 0.001	32.9 (17.8)	25.5 (20.9)	-7.4 (-11.2, -3.7)	< 0.001	-8.1 (-13.3 -2.9)	0.002	0.33
SSB, ounces; baseline consumption > 24oz	*n* = 223	50.9 (23.9)	27.9 (24.8)	-23.0 (-27.0, -19.0)	< 0.001	42.9 (16.3)	32.8 (22.5)	-10.3 ( -17.7, -2.9)	0.007	-12.7 (-21.2, -4.2)	0.003	0.45
**Body Mass Index (BMI )**												
BMI percentile, continuous	*n* = 461	73.0 (27.9)	73.7 (26.9)	0.66 (-0.2, 1.6)	0.156	75.0 (25.8)	75.7 (25.2)	0.71 (-1.3, 2.7)	0.492	-0.06 (-2.3, 2.2)	0.960	0.01
BMI z-score, continuous	*n* = 461	0.93 (1.1)	0.95 (1.11)	0.02 (-0.01, 0.05)	0.230	0.94 (1.06)	0.98 (1.01)	0.04 (-0.03, 0.11)	0.280	-0.018 (-0.09, 0.06)	0.639	0.05
BMI z-score; baseline SSB consumption > 8oz	*n* = 355	0.90 (1.14)	0.91 (1.12)	0.02 (-0.02, 0.06)	0.321	0.92 (1.10)	0.95 (1.05)	0.03 (-0.07, 0.12)	0.558	-0.008 (-0.11, 0.09)	0.879	0.02
BMI z-score; baseline SSB consumption > 12oz	*n* = 306	0.89 (1.15)	0.90 (1.13)	0.02 (-0.03, 0.08)	0.364	0.94 (1.10)	0.98 (1.03)	0.04 (-0.06, 0.14)	0.419	-0.016 (-0.12, 0.09)	0.776	0.04
BMI z-score; baseline SSB consumption > 24oz	*n* = 195	0.82 (1.19)	0.80 (1.17)	-0.01 (-0.09, 0.08)	0.863	0.75 (1.18)	0.86 (1.05)	0.11 (0.01, 0.22)	0.037	-0.12 (-0.26, 0.02)	0.087	0.25
**Quality of life (QOL)**												
School-related function^c^	*n* = 504	67.9 (17.6)	66.1 (18.8)	-1.7 (-3.9, 0.4)	0.114	67.2 (17.5)	63.4 (17.6)	-3.9 (-7.0, -0.7)	0.018	2.1 (-1.7, 6.0)	0.283	0.10
Overall health rating^d^	*n* = 521	3.5 (0.9)	3.6 (0.9)	0.06 (0.003, 0.1)	0.041	3.7 (0.9)	3.7 (0.8)	0.03 (-0.1, 0.2)	0.636	0.031 (-0.1, 0.2)	0.673	0.04

NOTE, some secondary outcomes (BMI and QOL) have reduced sample size due to missing data and outlier determination ≥ 2 × interquartile range cut-point.

^a^Means and Standard Deviations (SD) are not adjusted for covariates.

^b^Models control baseline covariates including gender, race, and degree of parent involvement. The models’ standard errors are adjusted to be school-year cohort cluster robust which is reflected in the 95% confidence intervals and *p*-values.

^c^Unit is percent (0-100), higher scores indicate better school-related quality of life function.

^d^Unit is 5-point Likert Scale (1–5), higher scores indicate better self-rated health.

Modified two-part models with fixed effects (e.g., survey year, school-year cohort, 7-month assessment time indicator, treatment group indicator, and interaction term between 7-month and treatment group) were used to estimate between group over time treatment effect for SSB primary outcomes. The modified two-part model was chosen to address the semi-continuous nature of reported SSB consumption [91]. SSB consumption was not an inclusion criterion for the trial; therefore, our sample contained modest numbers of zero SSB consumption reported. These “zeros” are true zero, instead of missing or censoring, and result in a highly skewed SSB outcome distribution. Also, to explore potential heterogenous treatment effects, SSB and BMI intervention effects were estimated on participants who had different thresholds of SSB consumption at baseline (i.e., > 8, >12 and > 24 SSB ounces/day). For other outcomes, generalized linear models with appropriate link function and family of distribution with similar specification were used. In addition, student models controlled for gender, race, and degree of caregiver engagement. Caregiver models controlled for gender and race. Covariates were identified a priori based on the literature and theory relevant to SSB behavior changes [92, 93]. All models’ standard errors were adjusted to be school-year cohort cluster robust which is reflected in the 95% confidence intervals and *p*-values. When examining missing patterns in 7-month outcomes, the likelihood of missing student SSB data was correlated with race, caregiver engagement, survey years, and treatment status. These were included in our models’ a priori chosen covariates [94]. Robustness of the completers analysis was examined by comparing them with intention-to-treat (ITT) multiple imputation approach. Missing 7-month SSB outcomes were imputed as a function of baseline SSB, gender, race, caregiver engagement (for student ITT), survey year, treatment status and school-year cohort. The imputer created 100 imputed data sets with negative imputed SSB values censored to zero to reflect the semi-continuous nature of the primary outcome. The final multiple imputation results combined all 100 sets of modified two-part model results into a set of pooled results accounting for the increased variability due to imputation following Rubin’s Rule [95].

Finally, descriptive statistics were used to summarize fidelity and perceived student engagement ratings. ANCOVA models controlled for schools and were used to explore differences between researcher and teacher ratings.

## Results

### Enrollment and retention

The study CONSORT diagram is illustrated in Fig. 1. Of 14 schools approached for trial participation, 12 agreed and were enrolled and randomized. One school district declined due to perceived sensitivity of student weight data collection, while the other did not respond to contact attempts. Of approximately 620 attending students from the 6 intervention schools, 357 (58%) enrolled in the study, of which 329 (92%) completed the 7-month follow-up. Of approximately 802 attending students from the 6 control schools, 308 (38%) enrolled in the study and 258 (84%) completed the follow-up. Additionally, 190 (31%) and 158 (20%) caregivers enrolled in the intervention and control arms, respectively. Of these, 126 (66%) intervention caregivers and 110 (70%) control caregivers completed the 7-month follow-up.

### Demographics

Of 526 students included in the analyses, the mean (SD) age was 12.7 (0.5) years and students were 55% female (Table 1). Notably, 46% of students were at an unhealthy weight, including 18% with overweight, 19% with obesity, and 9% with severe obesity.

Of 220 caregivers included in the analyses, the mean (SD) age was 40.6 (6.7) years (Table 2). Caregivers were mostly female (95%) and White (93%); 25% had a high school education or less and 33% lived in households with annual income less than $50,000. Also, 26%, 19%, and 25%, respectively, were with overweight, obesity, and severe obesity.

### Student outcomes

**Fig. 2:**
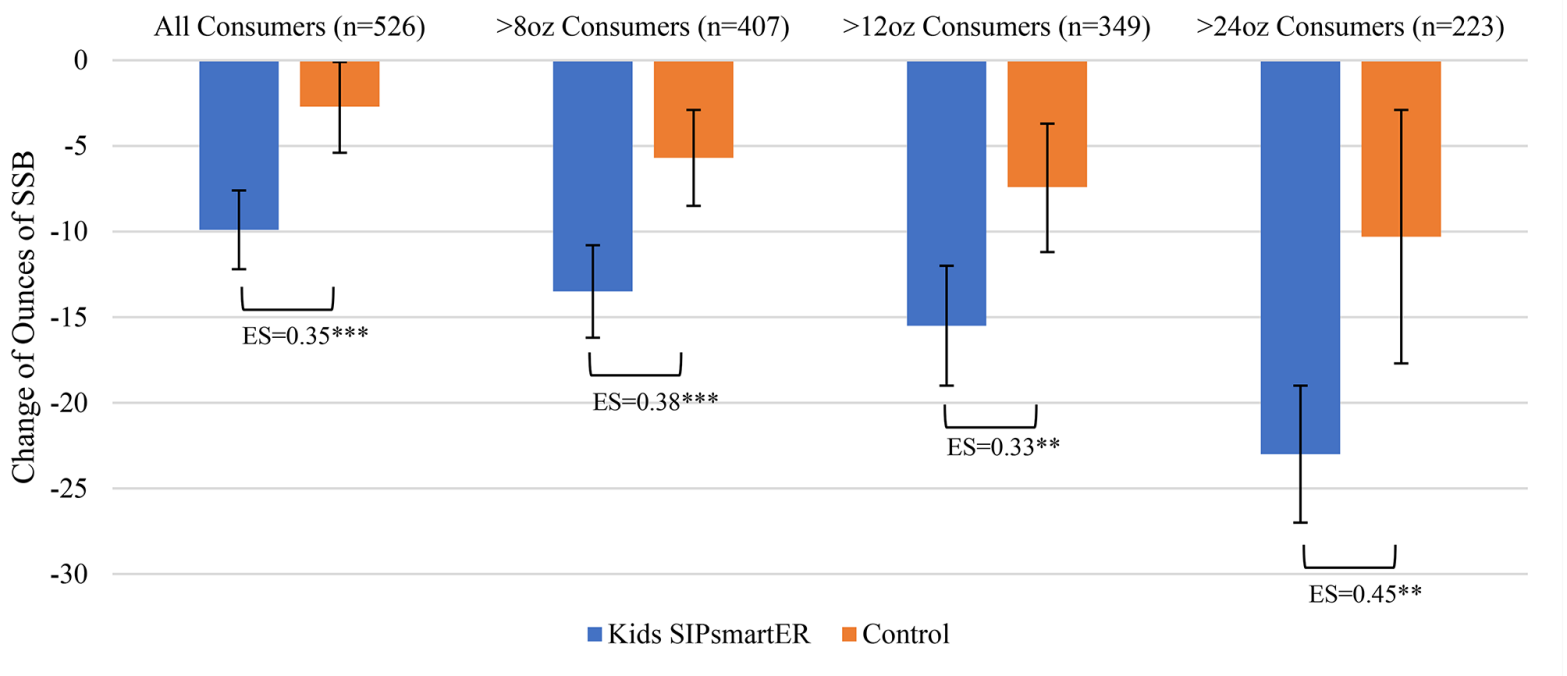
Student 0–7 month changes in SSB, by randomized condition and by consumption level at baseline ES = Effect size, *p < 0.05, **p < 0.01 ***p < 0.001

*SSB.* During the 7-month period, intervention students significantly decreased SSB by 9.9 ounces/day (95% CI = -12.2, -7.6; *p* < 0.001) compared to control students that decreased 2.7 ounces/day (95% CI=-5.4, -0.1; *p* = 0.045) (Table 3). Overall, intervention students’ SSB reduction averaged 7.2 ounces/day more than control students (*p* < 0.001), corresponding to a 0.35 ES. Among students who consumed > 8 (*n* = 407, 77%), > 12 (*n* = 349, 66%), and > 24 (*n* = 223, 42%) SSB ounces/day at baseline, ES increased to 0.38, 0.33, and 0.45, respectively (Table 3; Fig. 2).

**Fig. 3:**
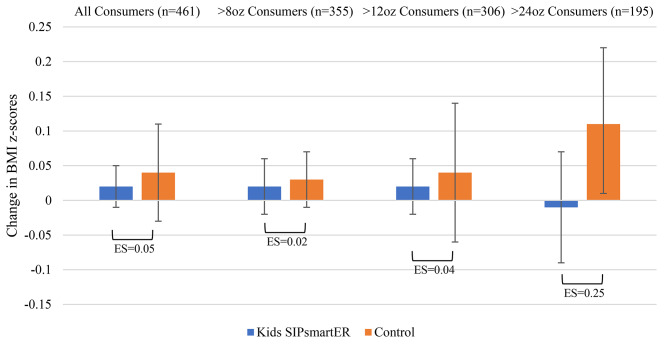
Student 0–7 month changes in BMI z-scores, by randomized condition and by consumption level at baseline ES = Effect size, *p < 0.05, **p < 0.01 ***p < 0.001

*BMI percentile and z-score.* When comparing intervention and control students, relative between group effects for BMI percentile (*p* = 0.645) and BMI z-scores (*p* = 0.639) were not statistically significant (Table 3). Among students who consumed > 24 SSB ounces/day at baseline (*n* = 195), there was a trend for intervention students’ average BMI z-score change − 0.12 units less than the control students (ES = 0.25, *p* = 0.087) (Table 3; Fig. 3).

*QOL and self-reported health.* No statistically significant relative between group effects were found for students’ school-related QOL (*p* = 0.283) or self-reported health (*p* = 0.673) (Table 3).

### Caregiver outcomes

**Fig. 4:**
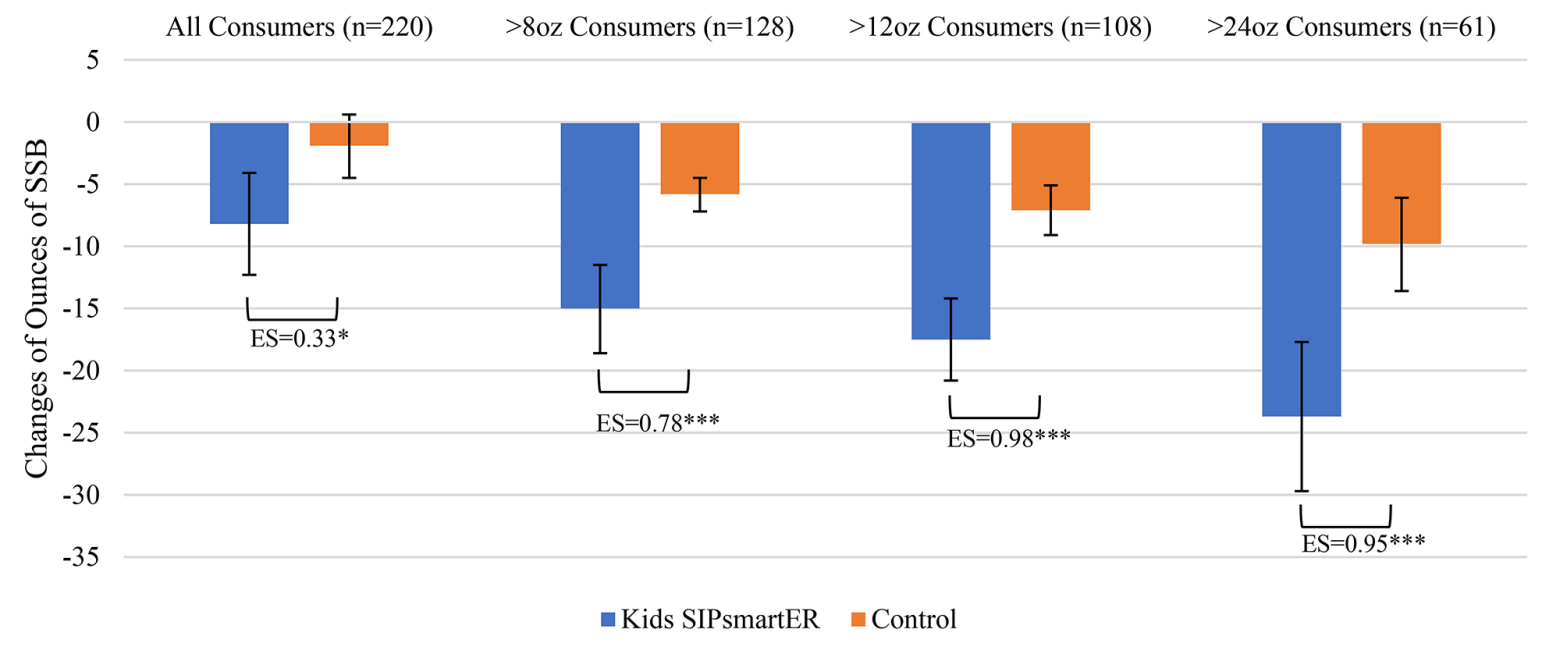
Caregiver 0–7 month changes in SSB, by randomized condition and by consumption level at baseline ES = Effect size, *p < 0.05, **p < 0.01 ***p < 0.001

*SSB.* Overall, intervention caregivers significantly decreased SSB by 8.2 ounces/day (95% CI = -12.3, -4.1; *p* < 0.001) relative to control caregivers with a non-significant decrease of 1.9 ounces/day (95% CI, -4.5, 0.6; *p* = 0.137) (Table 4). Intervention caregivers’ SSB reduction averaged 6.3 ounces/day more than control caregivers (*p* = 0.014), corresponding to a 0.33 ES. Among caregivers who consumed > 8 (*n* = 128, 58%), > 12 (*n* = 108, 49%), and > 24 (*n* = 61, 28%) SSB ounces/day at baseline, ES increased to 0.78, 0.98, and 0.95, respectively (Table 4; Fig. 4).

**Table 4:** Caregiver outcomes: 0–7 month changes in sugar-sweetened beverages (SSB), Body Mass Index (BMI) and quality of life (QOL), by randomized treatment condition

Variable	Sample size	Kids SIPsmartER				Control				Relative effects between conditions^b^		
		Baseline^a^	7-month^a^	Adjusted change baseline to 7-month^b^		Baseline^a^	7- month^a^	Adjusted change baseline to 7-month^b^				
		Mean (SD)	Mean (SD)	Coeff (95% CI)	p- value	Mean (SD)	Mean (SD)	Coeff (95% CI)	p- value	Coeff (95% CI)	p- value	Effect size
**Sugar-sweetened beverages (SSB)**												
SSB, ounces	*n* = 220	16.3 (18.0)	8.5 (12.9)	-8.2 (-12.3, -4.1)	< 0.001	17.0 (18.7)	14.6 (18.5)	-1.9 (-4.5, 0.6)	0.137	-6.3 (-11.3, -1.3)	0.014	0.33
SSB, ounces; baseline consumption > 8oz	*n* = 128	26.1 (17.4)	11.8 (15.1)	-15.0 (-18.6, -11.5)	< 0.001	28.5 (17.5)	22.1 (20.7)	-5.8 (-7.2, -4.5)	< 0.001	-9.2 (-13.2, -5.2)	< 0.001	0.78
SSB, ounces; baseline consumption > 12oz	*n* = 108	29.40 (17.4)	12.9 (16.3)	-17.5 (-20.8, -14.2)	< 0.001	31.4 (17.2)	23.9 (21.4)	-7.1 (-9.1, -5.1)	< 0.001	-10.4 (-14.5, -6.4)	< 0.001	0.98
SSB, ounces; baseline consumption > 24oz	*n* = 61	39.5 (17.1)	17.0 (19.7)	-23.7 (-29.7, -17.7)	< 0.001	42.6 (15.3)	32.3 (23.8)	-9.8 (-13.6, -6.1)	< 0.001	-13.9 (-21.5, -6.2)	< 0.001	0.95
**Weight and Body Mass Index (BMI )**												
Weight, pounds continuous	*n* = 198	196.8 (50.3)	195.4 (49.7)	-1.5 (-2.5, -0.4)	0.006	179.9 (54.6)	181.9 (54.0)	2.1 (0.2, 3.9)	0.033	-3.54 (-5.7, -1.4)	0.001	0.47
BMI, continuous	*n* = 197	32.0 (7.5)	31.7 (7.4)	-0.3 (-0.6, -0.1)	0.004	29.5 (8.1)	29.8 (8.1)	0.3 (-0.01, 0.6)	0.054	-0.65 (-1.0, -0.3)	0.001	0.47
BMI; baseline SSB consumption > 8oz	*n* = 111	32.2 (78)	32.0 (7.6)	-0.2 (-0.7, 0.3)	0.440	29.1 (8.7)	29.5 (8.5)	0.4 (-0.1, 0.9)	0.084	-0.60 (-1.3, 0.1)	0.085	0.32
BMI; baseline SSB consumption > 12oz	*n* = 92	32.2 (7.3)	31.9 (6.9)	-0.3 (-0.7, 0.1)	0.176	29.3 (8.2)	29.8 (8.1)	0.5 (-0.02, 0.97)	0.060	-0.75 (-1.4, -0.1)	0.020	0.49
BMI; baseline SSB consumption > 24oz	*n* = 51	33.1 (6.0)	32.9 (6.1)	-0.3 (-0.8, 0.2)	0.268	27.2 (6.7)	28.2 (7.1)	0.9 (0.6, 1.2)	< 0.000	-1.23 (-1.8, -0.6)	< 0.001	1.05
**Quality of life (QOL)**												
Number of unhealthy days^c^	*n* = 202	10.3 (10.2)	10.1 (10.4)	-0.3 (-1.1, 0.6)	0.541	10.0 (10.4)	8.8 (10.3)	-1.7 (-4.3, 0.9)	0.195	1.5 (-1.2, 4.2)	0.290	0.16
Overall health rating^d^	*n* = 212	3.19 (0.9)	3.2 (0.8)	0.04 (-0.0, 0.1)	0.311	3.4 (0.9)	3.3 (0.9)	-0.06 (-0.2, 0.1)	0.406	0.10 (-0.1, 0.3)	0.226	0.18

NOTE, some secondary outcomes (BMI and QOL) have reduced sample size due to missing data and outlier determination ≥ 2 × interquartile range cut-point.

^a^Means and Standard Deviations (SD) are not adjusted for covariates.

^b^Models control baseline covariates including gender and race. The models’ standard errors are adjusted to be school-year cohort cluster robust which is reflected in the 95% confidence intervals and *p*-values.

^c^Unit is number of unhealthy days in last 30 days, higher scores indicate worse QOL.

^d^Unit is 5-point Likert Scale (1–5), higher scores indicate better self-rated health.

**Fig. 5:**
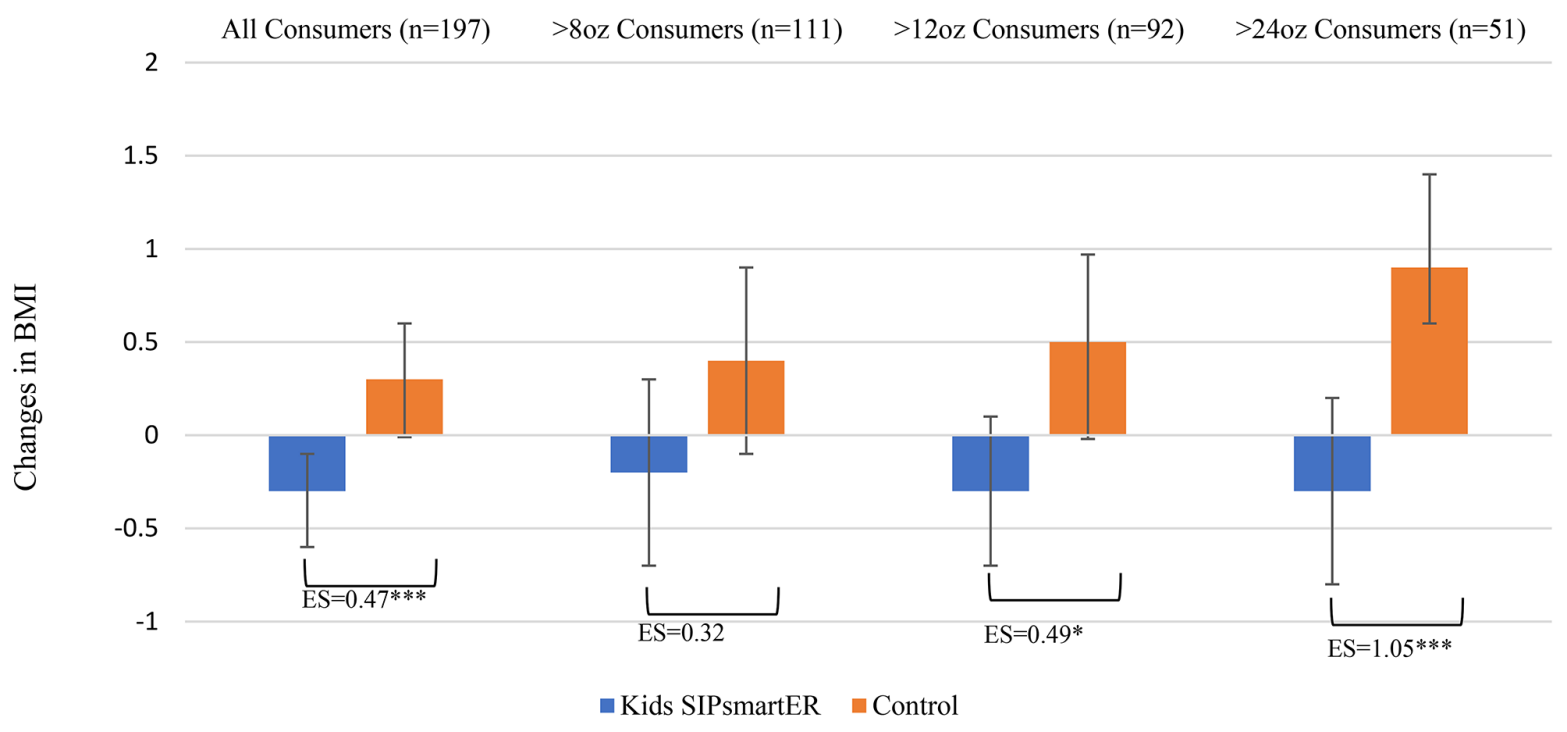
Caregiver 0–7 month changes in BMI, by randomized condition and by consumption level at baseline ES = Effect size, *p < 0.05, **p < 0.01 ***p < 0.001

*Weight and BMI.* Intervention caregivers’ self-reported weight significantly decreased by 1.5 pounds (95% CI = -2.5, -0.4; *p* = 0.004) compared to control caregivers who increased by 2.1 pounds (95% CI, 0.2, 3.9; *p* = 0.033) (Table 4). Intervention caregivers’ weight reduction averaged 3.5 pounds more than control caregivers (*p* = 0.001), corresponding to a 0.47 ES. Relative between group BMI ES (0.47) was similar (*p* = 0.001). Among caregivers who consumed > 12 (*n* = 92) and > 24 (*n* = 51) SSB ounces/day at baseline, BMI ES increased to 0.49 (*p* = 0.020) and 1.05 (*p* < 0.001, respectively (Table 4; Fig. 5).

*QOL and self-reported health.* Relative between group effects for caregivers’ self-reported number of unhealthy days (*p* = 0.290) and self-reported health (*p* = 0.226) were not statistically significant (Table 4).

### Completers versus Intention to treat analysis

Our completers and ITT analyses findings for SSB ounces, BMI, and QOL indicators were consistent in terms of ES, directions, and statistical significance. Yet, as expected, the ITT approach introduced more computation noise, faced larger variations, and therefore produced less precise estimations. Also, compared to students, the caregiver sample was relatively smaller with a higher proportion of missing SSB data and thus had reduced precision on SSB outcomes. For example, the ITT analyses revealed significant Kids SIP*smart*ER intervention effects for the primary SSB ounces outcome, yet with a decreased ES to 0.22 (*p* = 0.013) among students and 0.25 (*p* = 0.023) among caregivers.

### Intervention Fidelity

Of 12 planned lessons at the 6 intervention schools, 100% (72 of 72) were delivered. Fidelity checklists were completed by 100% and 96% of researchers and teachers, respectively. Overall, fidelity ranked high at 96.7% (SD = 4.3%) with significantly higher ranks among teachers [99.7% (SD = 1.0%)] relative to researchers [95.0% (SD = 4.6%)] (*p* = 0.016). As identified by researchers, ∼ 3% of lesson specific activities were modified to meet unique circumstances in given class periods. Rated perceptions of student engagement were also high, averaging 6.2 (SD = 0.7) out of 7, with significantly higher ratings among teachers [6.6 (SD = 0.4)] compared to researchers [6.0 (SD = 0.7)] (*p* = 0.029). Also, Qualtrics reports indicated that researchers sent 100% of caregiver text messages as intended. Qualtrics message distribution reports revealed about 2% of caregivers did not receive text messages due to non-functioning phones.

## Discussion

Kids SIP*smart*ER was effective at reducing SSB among students and their caregivers in the rural, medically underserved Appalachian region. Our trial addresses notable gaps in the SSB intervention literature [40–42] and our findings are largely consistent with a recent systematic review of school-based trials that found promising results in reducing SSB among adolescents using educational/behavior interventions [40]. Notably, our SSB ES for all enrolled participants [i.e., student ES = 0.35 (*p* < 0.001), caregiver ES = 0.33 (*p* = 0.014)] were substantially larger than a recent RCT meta-analysis of SSB reduction interventions [i.e., five reviewed adolescent studies SSB ES = 0.05 (*p* = 0.04), 12 reviewed adult studies SSB ES = 0.07 (*p* = 0.16)] [41]. More specifically, the approximate 6–7 ounces/day decrease in SSB among all students and caregivers and approximate 13–14 ounces/day decrease in SSB among students and caregivers who were the highest consumers is clinically significant [18–28, 96]. These findings highlight the promise of Kids SIP*smart*ER as a primary prevention intervention to reduce SSB consumption. Similarly, from a public health perspective, results emphasize the value in creating awareness and providing SSB intervention strategies regardless of current SSB behaviors. It is difficult to say with certainty which components of Kids SIP*smart*ER contribute to its effectiveness, yet in alignment with systematic review findings of school-based and adolescent-focused SSB interventions, we suspect that the regulatory behavioral change techniques (e.g., action plan development, goal setting, SSB self-monitoring) and efforts to involve parents as role models and social support contributed to its success [40, 43].

Given the primary and secondary prevention framing and school-based setting of Kids SIP*smart*ER, all students and caregivers were eligible to participate, regardless of baseline SSB consumption. Nonetheless, among all enrolled students, baseline SSB averaged 30 ounces/day and 42% consumed > 24 ounces/day. Likewise, among all enrolled caregivers, baseline SSB averaged 16 ounces/day and 28% consumed > 24 ounces/day. While some U.S. reports indicate a decline in SSB [8], students and caregivers in our study are consuming substantially higher SSB amounts relative to national U.S. estimates [9, 12]. Importantly, our SSB effects were even stronger among students and caregivers who were high SSB consumers at baseline, further underscoring the value of Kids SIP*smart*ER as a school-based intervention targeting rural counties where SSB behavioral and related health disparities persist.

Involving middle school caregivers in a school-based behavioral intervention presents both opportunities and challenges. Established ecological models highlight the role of caregivers and the home environment in child health and obesity [97, 98], including for SSB-specific behaviors [32–37]. Two cross-sectional adolescent studies, one in a U.S. national sample and one in an Appalachian sample, both demonstrate caregivers’ SSB rules and practices and the home environment as the strongest predictors of adolescent SSB intake [38, 39]. However, struggles with caregiver engagement in school-based health promotion programs are well documented, particularly among caregivers of adolescent students [99]. In recent years, text messaging has emerged as an effective intervention strategy [57–59]. Two text message intervention meta-analyses, across a wide range of behaviors, have shown aggregated ES of 0.39 (*p* < 0.001) among 19 RCTs [57] and 0.24 (*p* < 0.001) among 35 pre-post design studies (with or without a control group) [59]. Our 0.33 ES for SSB among caregivers is in alignment with these meta-analyses. Additional baseline, process, and engagement data from our text messaging intervention is published elsewhere [77, 100]. Gaps in school-based text message intervention literature targeting middle school caregivers limit our ability to directly compare to other similar studies, yet also highlights the unique contribution of our study, especially within the context of schools in medically underserved rural regions [101, 102].

As secondary outcomes, student height and weight were objectively measured while caregiver height and weight were self-reported. Since neither SSB nor BMI were inclusion criterion, we did not have expectations that BMI would be significantly impacted in a 7-month primary prevention intervention. Indeed, there were no significant within or between condition BMI changes among students. Yet, among students consuming > 24 ounces/day there was an interesting trend whereby Kids SIP*smart*ER students BMI z-score appear to stabilize, on average, over the 7-month period, while the control students BMI z-scores trend upward. Exploring heterogeneity of treatment effects by baseline SSB consumption in a primary prevention intervention study is an important contribution of our study [28]; however, future fully powered studies are needed to further investigate this trend. Also, a significant treatment effect on caregivers’ weight was detected, in favor of Kids SIP*smart*ER. Given known issues with self-reported weight, these data should be cautiously interpreted. Nonetheless, our RCT design provided a signal for weight reduction among caregivers receiving the Kids SIP*smart*ER intervention. Epidemiological data, including high-quality systematic reviews, demonstrate relationships between SSB and weight among adolescents and adults [20, 21, 28, 103, 104]. Specific to children and adolescents, a recent meta-analysis and dose response analysis of 121,282 participants found that high SSB intake was associated with a 0.75 unit (kg/m^2^) increase in BMI [20]. Also, a 17-year birth cohort found that for each additional 8 SSB ounces/day consumed throughout childhood and adolescence, BMI z-scores significantly increased by an average of 0.05 units, even when adjusting for energy intake and baseline socioeconomic status [96]. While we were not able to adjust for energy intake in our study, our intervention findings provide additional clinical evidence on relationships between SSB and weight [28]. Understanding the impacts of SSB changes on weight outcomes over a longer period is an important future endeavor.

Quality of life outcomes provide a participant-centered check, indicator of unintended negative consequences, and is a key outcome for effectiveness trials conducted in real-world settings [105]. Lack of between group differences in our samples imply that Kids SIP*smart*ER did not negatively impact students’ school-related function and caregivers’ number of unhealthy days. Yet, findings also indicate the intervention did not improve QOL or overall self-rated health as compared to the control condition. In a review of 55 SSB trials for children and adolescents, only 5% reported QOL or unintended consequences, making comparisons to the broader literature difficult [42].

Several key limitations should be considered when interpreting findings. First, due to the unique rural Appalachian region, our study may only be generalized to regions with similar cultural norms and disparities. Second, uncontrollable study disruptions caused by COVID-19 should be acknowledged, most notably the varied methods of survey data collection at some schools. Our statistical methods (i.e., controlling for survey years and assessment time fixed effects and addressing school-year cluster in inferences) help mitigate, but do not completely resolve, this concern. Also, halting enrollment of new schools in the 2020–2021 academic year, during the height of COVID-19 when most schools were remote and/or hybrid, helped promote internal validity of our study and ensured consistent intervention implementation across all schools– a finding further supported by our high fidelity ratings. Still, retention rates were clearly lower in spring of 2020 when schools were forced to close due to COVID-19, and enrollment rates were notably lower in 2021–2022 when schools were transitioning back and still closing regularly due to outbreaks. Third, as previously mentioned, limitations of caregivers’ self-reported weight should be considered, even though this concern is minimized by the RCT design. Fourth, our trial was not specifically powered to examine potential heterogenous treatment effects; thus, SSB and BMI intervention effects by thresholds of SSB consumption at baseline should be interpreted with caution. Finally, manualized recruitment and data collection protocol were applied at each school; however, research staff were not blinded to schools’ randomized allocation which has the potential of introducing bias [106]. These limitations should be interpreted with our study’s strengths, including a well-designed and successfully executed cluster RCT, robust theory-guided intervention targeting both students and caregivers, use of validated measures and present at follow-up and ITT analytical approaches, and focus on a medically underserved rural region with known SSB-related disparities.

Our on-going trial is currently focused on maintenance of behaviors and sustainability of Kids SIP*smart*ER among enrolled schools. Other planned analyses will further inform next steps [60], including (1) effects on secondary student and caregiver outcomes (e.g., other beverages; theoretical, health literacy, and parenting practice outcomes) and 18-month maintenance outcomes, (2) school-level organizational outcomes (e.g., implementation fidelity when teachers deliver Kids SIP*smart*ER, principal and teachers perceptions), and (3) differences in effectiveness data when Kids SIP*smart*ER is co-delivered by researchers-teachers versus when delivered by teachers only. When available, these additional findings will be shared with school decision makers to inform future directions, including a potential scale-up dissemination study. Finally, other promising approaches should be considered, including efforts to examine feasibility and impact of enhancing Kids SIP*smart*ER with higher-level environmental strategies [107] and directly texting students with tailored behavioral strategies to support adherence to SSB recommendations [108, 109].

## Conclusions

In conclusion, despite COVID-related study disruptions, Kids SIP*smart*ER was effective at reducing SSB consumption among Appalachian students and their caregivers. Importantly, SSB effects were even stronger among students and caregivers who were high SSB consumers at baseline. Our trial fills important literature gaps on an SSB-focused primary prevention school-based intervention conducted in a rural and historically underserved region, including use of text messaging as a caregiver intervention strategy. When available, additional trial data will be interpreted alongside the promising SSB effectiveness data to inform external validity and potential long-term sustainability of Kids SIP*smart*ER within schools.

## Data Availability

The datasets generated during the current study are available from the corresponding author on reasonable request.

## Abbreviations

SSB: sugar-sweetened beverages
BMI: body mass index
QOL: quality of life
RE-AIM: reach, adoption, effectiveness, implementation, and maintenance
TPB: Theory of Planned Behavior
RCT: randomized controlled trial
CDC: Centers for Disease Control
ITT: intention-to-treat
